# Factors Influencing Quality of Life in Hemodialysis Patients With Chronic Kidney Disease at a Tertiary Care Hospital in Eastern India

**DOI:** 10.7759/cureus.88740

**Published:** 2025-07-25

**Authors:** Rajdeep Shaw, Sanjoy K Sadhukhan, Jayita Pal, Pinaki Mukherjee, Aniruddha Biswas

**Affiliations:** 1 Epidemiology and Public Health, All India Institute of Hygiene and Public Health, Kolkata, IND; 2 Nephrology, Nil Ratan Sircar Medical College and Hospital, Kolkata, IND

**Keywords:** comorbidity, hemodialysis, kidney disease quality of life, kolkata, socioeconomic factors

## Abstract

Background

Chronic kidney disease (CKD) is a growing health burden worldwide, often requiring hemodialysis to sustain life in advanced stages. This study evaluates the quality of life (QoL) and its determinants among CKD patients undergoing hemodialysis at a tertiary care hospital in Kolkata, focusing on sociodemographic, clinical, and psychological factors to facilitate better patient-centered care in resource-limited settings.

Methodology

A hospital-based, cross-sectional study was conducted among 228 CKD patients on maintenance hemodialysis at a tertiary care center in Kolkata. QoL was assessed using the validated Kidney Disease Quality of Life-Short Form (KDQOL-SF™) questionnaire, alongside sociodemographic and clinical factors. Systematic random sampling and face-to-face interviews were employed for data collection. Collected data were entered in Microsoft Excel (Microsoft Corp., Redmond, WA, USA) and subsequently analyzed in Jamovi software.

Results

The study reported a moderate mean QoL score (49.96 ± 14.50), with 134 (58.7%) patients experiencing fair to poor QoL. Poor QoL was significantly associated with low socioeconomic status (adjusted odds ratio (AOR) = 2.34, p = 0.005), presence of comorbidities (AOR = 3.79, p < 0.001), and CKD duration exceeding five years (AOR = 3.23, p < 0.001), though patients undergoing fewer than 100 dialysis sessions annually had significantly lower odds of poor QoL (AOR = 0.34, p < 0.001).

Conclusions

QoL in CKD patients on hemodialysis is markedly compromised, with low socioeconomic status, comorbidities, prolonged disease duration, and dialysis frequency independently influencing outcomes. Targeted strategies to improve dialysis adherence, comprehensive comorbidity management, and the reduction of socioeconomic barriers are essential to enhance patient well-being and overall QoL.

## Introduction

Chronic kidney disease (CKD) is a progressive condition marked by irreversible loss of kidney function, potentially leading to end-stage renal disease (ESRD) [1]. Globally, CKD affects approximately 9-13% of the population, contributing significantly to morbidity and mortality, especially in low- and middle-income countries (LMICs) [2]. In India, the prevalence of CKD is increasing due to common risk factors such as hypertension, diabetes, and age-related degeneration [3]. As kidney function deteriorates, the body loses its ability to eliminate toxins and maintain fluid balance, necessitating renal replacement therapies such as dialysis to sustain life [4].

Hemodialysis is the most widely used renal replacement therapy, functioning through extracorporeal removal of waste and excess fluid [5]. While hemodialysis helps manage symptoms and extend survival in ESRD patients, it imposes considerable physical, emotional, and social burdens. Patients often face fatigue, infections, cardiovascular complications, and dietary and lifestyle restrictions, all of which significantly impact their overall quality of life (QoL) [6-8].

QoL is a broad concept encompassing physical, psychological, and social well-being [9]. In chronic illnesses such as CKD, where treatment is continuous and demanding, QoL becomes a vital indicator of health beyond clinical outcomes [10]. Hemodialysis patients frequently report poor QoL due to physical limitations, emotional distress, and reduced participation in social and occupational roles [11-13]. These impairments highlight the need for patient-centered care approaches that evaluate not just survival but also the lived experience.

Studies in developed countries have shown that integrating psychosocial support with standard medical care can enhance QoL among hemodialysis patients [14,15]. However, despite the increasing burden of CKD in India, limited research has explored QoL among hemodialysis patients, especially in eastern India and Kolkata. Most existing Indian studies are concentrated in southern regions or major metropolitan centers and tend to focus primarily on clinical or demographic factors [16]. Furthermore, the interplay of psychological and sociodemographic factors such as depression, anxiety, education level, income, and social support remains underexplored in these populations [17-20].

## Materials and methods

Study design and setting

A hospital-based, cross-sectional study was conducted from January 2023 to December 2025 in the Nephrology Department of Nil Ratan Sircar Medical College and Hospital, Kolkata. According to department records, an average of 100 patients (including both new and follow-up patients) undergo hemodialysis each week.

Study participants and selection criteria

The study population included all adult CKD patients undergoing hemodialysis at the hospital’s Nephrology Department. Patients who had been on maintenance hemodialysis for more than three months were included. Critically ill patients and those who did not provide informed consent were excluded from the study.

Sample size and sampling technique

A prior study conducted in Chennai in 2013 using the Kidney Disease Quality of Life-Short Form (KDQOL-SF™) questionnaire reported a mean physical domain score of 25 with a standard deviation (SD) of 11.85 [21,22]. Based on these parameters, using Cochran’s formula with a 95% confidence level and 2 units of precision, the required sample size was calculated to be 135. After adjusting for a design effect of 1.5 and a 10% non-response rate, the final sample size was estimated to be 228.

Systematic random sampling was employed to select participants from the Nephrology outpatient (OPD) register. Data collection took place weekly on Fridays over 40 weeks, enrolling six patients per day. With approximately 100 patients visiting the OPD daily, every 17th patient was selected after a randomly chosen starting point. In cases of non-consent, the next eligible patient was approached. Each participant was assigned a unique ID. Data collection included interviews, anthropometric measurements, blood testing, and review of medical records.

Study tools and data collection techniques

The data collection tool was a predesigned, pretested, semi-structured questionnaire comprising sections on sociodemographic details, behavioral patterns, clinical characteristics, and QoL. The KDQOL-SF™ Version 1.3 was used to assess QoL, a validated tool specifically designed for CKD patients undergoing dialysis [22]. It includes both general health and disease-specific domains, scored on a 0-100 scale where higher scores indicate better QoL. Composite scores summarize physical and mental health status.

The questionnaire was developed with input from public health experts, ensuring relevance and comprehensiveness. Initially drafted in English, it was translated into Bengali and Hindi, and then back-translated to preserve semantic integrity. The language was simplified for patient comprehension. Pre-testing was conducted on 30 CKD patients from a different facility to assess reliability and clarity. Necessary revisions were incorporated before finalization.

Data were collected through face-to-face interviews, followed by anthropometry, hemoglobin testing, and review of clinical records after obtaining informed written consent.

Ethical considerations

Ethical clearance was obtained from the Institutional Ethics Committee of both the All India Institute of Hygiene and Public Health, Kolkata, and Nil Ratan Sircar Medical College and Hospital, Kolkata. Informed written consent was taken from all participants before inclusion in the study.

Statistical analysis

Data were entered into Microsoft Excel 2016 (Microsoft Corp., Redmond, WA, USA), cleaned, coded, and analyzed using Jamovi software. Descriptive statistics such as frequencies, percentages, means, SDs, medians, interquartile ranges (IQRs), and ranges were computed and presented in tables. Normality of data was assessed using the Kolmogorov-Smirnov and Shapiro-Wilk tests, as well as histogram inspection.

Chi-square tests were used to determine associations between QoL and independent variables. QoL scores were dichotomized at the median value into high QoL (≥50) and low QoL (<50). Odds ratios (ORs) with 95% confidence intervals (CIs) were calculated. Variables significant in bivariate analysis were included in a multivariable logistic regression model to determine independent predictors of QoL. All statistical tests were two-tailed, with a significance threshold of p < 0.05.

## Results

Sociodemographic and behavioral characteristics

The median age of the study participants was 42 years (IQR = 35-48 years), with ages ranging from 18 to 73 years. Approximately 35% of the participants were in the 46-60-year age group. Of the 228 patients with CKD, 60.1% were male. A majority (79.8%) of the participants were married. Regarding the place of residence, 60.1% lived in urban areas. In terms of family structure, 70.2% of the participants belonged to nuclear families. Concerning educational status, 20.2% were illiterate, 25% were educated up to the primary level, and 35.1% had completed secondary education. With respect to occupation, 25% of participants were engaged in skilled occupations, while 20% were unemployed. Socioeconomically, 30.3% of participants were classified under the middle class (Class III) according to the Modified BG Prasad Scale (2024). Substance use history revealed that 50% of participants had used substances, while the remaining 50% had never used any substances. Among the substance users (n = 114), 59.6% were currently using some form of substance, while the rest had discontinued use. The most commonly reported substance was smokeless tobacco (79.8%), followed by smoking tobacco (59.6%) (Table 1).

**Table 1:** Distribution of study participants according to sociodemographic and behavioral characteristics (n = 228). SD = standard deviation; IQR = interquartile range

Variable	Frequency (%)
Age group (years)	
18–30	34 (14.9)
31–45	57 (25)
46–60	80 (35.1)
>60	57 (25)
Median (IQR) = 42 (35.48)	
Gender	
Male	137 (60.1)
Female	91 (39.9)
Marital status	
Currently married	182 (79.8)
Never married	23 (10.1)
Widowed	18 (7.9)
Divorced/separated	5 (2.2)
Residence	
Rural	91 (39.9)
Urban	137 (60.1)
Type of family	
Nuclear	160 (70.2)
Joint	68 (29.8)
Educational status	
Illiterate	46 (20.2)
Primary	57 (25.0)
Secondary	80 (35.1)
Graduate and above	45 (19.7)
Occupation	
Unemployed	46 (20.2)
Retired	34 (14.9)
Skilled	57 (25)
Semiskilled	46 (20.2)
Unskilled	45 (19.7)
Socioeconomic status (based on the modified BG Prasad scale)	
Upper	23 (10.1)
Upper middle	46 (20.2)
Middle	69 (30.3)
Lower middle	57 (25)
Lower	33 (14.5)
Mean (SD) = INR 2,955.72 (843); range = INR 1,082–18,756	
Substance use	
Ever used	114 (50)
Never used	114 (50)
Ever used (n = 114)	
Currently using	68 (59.6)
Quit	46 (40.4)
Types of substance use (n = 114)	
Smoking tobacco	68 (59.6)
Smokeless tobacco	91 (79.8)
Alcohol	57 (50)

Disease profile

Among the 228 patients with CKD, 76.3% had associated comorbidities. The most common comorbidity was hypertension (74.7%), followed by diabetes mellitus (51.1%). Half of the CKD patients (50%) were diagnosed with the disease between the ages of 41 and 60 years. Regarding disease duration, 45.2% of the participants had been living with CKD for 1-5 years. In terms of dialysis history, 39.9% of the participants had undergone hemodialysis for 1-3 years, with a mean duration of 5.2 ± 2.1 years. Additionally, 45.2% of the patients received between 101 and 150 sessions of hemodialysis per year (Table 2).

**Table 2:** Distribution of study participants according to the clinical and disease profile (n = 228). CKD = chronic kidney disease; T2DM = type 2 diabetes mellitus; BMI = body mass index

Variables	Frequency (%)
Disease profile	
Comorbidity	
Present	174 (73.6)
Types of comorbidities	
Hypertension	130 (57)
T2DM	89 (39)
Thyroid disease	35 (15.3)
Family history of CKD	
Present	46 (20.2)
Age of diagnosis (years) of CKD	
<20	11 (4.8)
20–40	46 (20.2)
41–60	114 (50)
>60	57 (25)
Duration of illness (years)	
<1	34 (14.9)
1–5	103 (45.2)
6–10	68 (29.8)
>10	23 (10.1)
Duration of hemodialysis (years)	
<1	40 (17.5)
1–3	91 (39.9)
4–6	68 (29.8)
>6	29 (12.7)
Frequency of hemodialysis sessions per year	
<50	23 (10.1)
50–100	80 (35.1)
101–150	103 (45.2)
>150	22 (9.6)
Clinical profile	
Anemia (WHO classification)	
Present	176 (77.2)
Absent	52 (22.8)
BMI (WHO classification)	
Undernutrition (<18.5 kg/m^2^)	34 (14.9)
Normal (18.5–24.9 kg/m^2^)	98 (43)
Overweight (25–29.9 kg/m^2^)	65 (28.5)
Class I Obesity Class I Class II Class III	21 (9.2)
Class II Obesity	7 (3.1)
Class III Obesity	3 (1.3)

Clinical profile

Among the 228 CKD patients, 77.2% had anemia. A normal body mass index (BMI) was observed in 43% of participants, while 28.5% were classified as overweight (Table 2).

Quality of life scores (overall and domain-wise)

The mean overall QoL score among the participants was 49.96 ± 14.50. The mean physical health composite score was 52.4 ± 12.1, which included domains related to general health, physical functioning, bodily pain, and role-physical limitations. The mean mental health composite score was 58.6 ± 13.4, encompassing emotional well-being, role-emotional functioning, social functioning, and energy/fatigue. In addition, kidney disease-specific domains addressed aspects unique to CKD, such as the perceived impact of the disease and patient satisfaction with care and symptoms. Among these, the patient satisfaction domain recorded the highest mean score at 65.8 ± 12.2, followed by staff encouragement during dialysis, which had a mean score of 62.3 ± 13.4 (Table 3). With more than half of the study population (58.7%) experiencing fair to poor QoL, indicating an average QoL with moderate impairments to significant challenges in health and well-being. Each domain was assessed on a 0-100 scale, where higher scores indicated better QoL. The QoL evaluation was categorized into three major components (Table 4).

**Table 3:** QoL responses (scores) by KDQOL-SF Scale domain-wise. QoL = quality of life; KDQOL-SF = Kidney Disease Quality of Life-Short Form; SD = standard deviation; IQR = interquartile range

Domains	Mean	SD	Median	IQR	Range
Physical health composite	52.4	12.1	52	40–65	30–80
General health perception	45.8	10.9	45	34–54	20–75
Physical functioning	48.2	14.7	48	33–63	25–90
Pain	50.7	15.3	51	35–60	20–85
Role-physical	47.9	13.2	48	35–61	25–80
Mental health composite	58.6	13.4	57	45–72	35–90
Emotional well-being	59.3	12.8	58	45–63	30–85
Role-emotional	57.8	13.9	57	41–73	35–85
Social functioning	55.1	14.5	55	40–70	25–85
Energy/Fatigue	53.4	11.6	53	41–65	25–75
Cognitive functioning	61.0	12.3	61	50–70	40–85
Burden of kidney disease	39.5	13.5	39	25–55	15–70
Symptoms/Problem list	51.8	11.2	52	40–68	30–80
Effects of kidney disease	47.4	12.9	47	35–63	25–75
Work status	35.7	14.6	36	20–56	10–70
Dialysis staff encouragement	62.3	13.4	62	47–80	40–85
Patient satisfaction	65.8	12.2	65	50–81	50–90

**Table 4:** QoL responses (scores) by KDQOL-SF scale. QoL = quality of life; KDQOL-SF = Kidney Disease Quality of Life-Short Form

Category	Score range	Frequency (n)	Percentage (%)
Excellent	81–100	25	11.0
Good	61–80	62	27.2
Fair	41–60	89	39.0
Poor	21–40	45	19.7
Very poor	0–20	7	3.1

Determinants of quality of life

In the multivariable logistic regression analysis, several factors were identified as significant predictors of poor QoL among CKD patients. Patients from lower socioeconomic backgrounds had significantly higher odds of reporting poor QoL (adjusted odds ratio (AOR) = 2.34, p = 0.005). The presence of comorbidities was also strongly associated with poor QoL, with affected individuals being nearly four times more likely to have low QoL (AOR = 3.79, p < 0.001). Additionally, a longer duration of CKD exceeding five years significantly increased the likelihood of poor QoL (AOR = 3.23, p < 0.001). Patients receiving fewer than 100 hemodialysis sessions per year had significantly lower odds of poor QoL (AOR = 0.34, p < 0.001), suggesting that more frequent dialysis may contribute to better QoL outcomes. On the other hand, age, gender, substance use, and age at diagnosis did not retain statistical significance in the adjusted model. However, substance use showed a marginal association with poor QoL (AOR = 1.82, p = 0.052), indicating a potential trend that warrants further investigation. The Hosmer-Lemeshow goodness-of-fit test yielded a p-value greater than 0.05, indicating a good fit of the logistic regression model to the observed data. The Nagelkerke R² value was estimated at 0.37, suggesting that the variables included in the model explained approximately 37% of the variance in QoL outcomes (Table 5).

**Table 5:** Determinants of QoL among the study participants (n = 228). CKD = chronic kidney disease; QoL = quality of life; OR = odds ratio; CI = confidence interval

Variable	Low QoL n (%)	OR (95% CI)	P-value	Adjusted OR (95% CI)	P-value
Age group (years)					
≤50 (n = 81)	25 (30.9%)	0.59 (0.32–1.08)	0.040*	0.67 (0.35–1.23)	0.140
>50 (n = 147)	72 (49.0%)	Reference	-	Reference	-
Gender					
Male (n = 140)	60 (42.9%)	1.04 (0.61–1.76)	0.940	1.12 (0.65–1.92)	0.680
Female (n = 88)	37 (42.0%)	Reference	-	Reference	-
Socioeconomic status					
Low (BG Prasad IV and V) (n = 120)	65 (54.2%)	2.82 (1.65–4.84)	0.001**	2.34 (1.28–4.27)	0.005**
High (BG Prasad I–III) (n = 108)	32 (29.6%)	Reference	-	Reference	-
Presence of comorbidities					
Present (n = 142)	78 (54.9%)	4.19 (2.29–7.68)	<0.001**	3.79 (1.91–7.52)	<0.001**
Absent (n = 86)	19 (22.1%)	Reference	-	Reference	-
Substance usage					
Present (n = 94)	50 (53.2%)	2.08 (1.19–3.64)	0.011*	1.82 (0.99–3.33)	0.052
Absent (n = 134)	47 (35.1%)	Reference	-	Reference	-
Age at diagnosis of CKD (years)					
≤50 (n = 100)	35 (35.0%)	0.56 (0.32–0.97)	0.042*	0.69 (0.38–1.26)	0.210
>50 (n = 128)	62 (48.4%)	Reference	-	Reference	-
Duration of CKD (years)					
≤5 (n = 130)	38 (29.2%)	0.27 (0.16–0.47)	<0.001**	0.31 (0.18–0.55)	<0.001**
>5 (n = 98)	59 (60.2%)	Reference	-	Reference	-
Frequency of hemodialysis					
≤100 sessions/year (n = 138)	43 (31.2%)	0.27 (0.15–0.47)	<0.001**	0.34 (0.18–0.61)	<0.001**
>100 sessions/year (n = 90)	54 (60.0%)	Reference	-	Reference	-

## Discussion

The present study assessed the QoL and its determinants among CKD patients undergoing hemodialysis at a government tertiary care hospital in Kolkata. Key findings of the study revealed that a substantial proportion of patients experienced poor QoL, particularly in domains related to physical functioning and emotional well-being. Psychological factors such as depression and anxiety emerged as significant determinants, along with sociodemographic variables including income, education, and social support.

CKD is a progressive and debilitating condition that adversely affects multiple dimensions of patient health, particularly among individuals undergoing hemodialysis. This study evaluated the QoL of CKD patients using the KDQOL-SF™ scale and explored key determinants influencing QoL outcomes. The findings offer critical insights for improving comprehensive patient management.

Overall quality of life

The mean QoL score of the study population was 49.96 ± 14.50, indicating moderate impairment. This is consistent with previous research showing that dialysis patients have substantially lower QoL than the general population [23,24]. Factors such as physical symptoms, dietary and fluid restrictions, financial burden, and psychological distress contribute significantly to reduced QoL [25]. In this study, 42.5% of patients reported poor QoL, comparable to findings from other studies in LMICs like India, where resource constraints and economic burdens have been shown to adversely impact patient outcomes [26]. In contrast to developed nations, patients in LMICs frequently face limited dialysis access, minimal psychosocial support, and out-of-pocket healthcare expenditures, all of which contribute to poorer QoL [27].

Association with age

Although older participants (>50 years) in this study tended to report lower QoL scores, the association was not statistically significant after adjustment. This is consistent with studies indicating that age is not a strong independent predictor of QoL among hemodialysis patients [10,17]. For example, Mapes et al. observed that dialysis adequacy and comorbidities had a greater impact on QoL than chronological age [10]. Similarly, Mujais et al. emphasized the influence of clinical and psychosocial factors over age alone [17]. While aging may impact physical domains, overall QoL is determined by a combination of interrelated factors [28].

Gender differences

No significant difference in QoL was observed between male and female patients in this study. This aligns with earlier findings indicating that gender does not independently influence QoL after controlling for clinical variables [10,17]. Although women may experience higher rates of psychological symptoms, these do not consistently translate into differences in global QoL scores [29]. Therefore, gender alone may not be a critical determinant of QoL in patients receiving hemodialysis.

Socioeconomic status

Low socioeconomic status was significantly associated with poor QoL, with patients from BG Prasad Classes IV and V showing 2.34 times higher odds of reporting low QoL. Similar findings have been documented in other LMICs. Anees et al. found that lower income levels significantly impaired QoL among hemodialysis patients in Pakistan [16], while Lemos et al. reported socioeconomic status as a key QoL determinant in Brazil [12]. In India, studies by Murali et al. and Bohlouli et al. also confirmed that socioeconomic disadvantage is a major barrier to optimal health and well-being in CKD patients [21,24]. These results underscore the urgent need for financial and social support mechanisms in CKD care.

Comorbidities

Comorbid conditions emerged as the most significant predictor of poor QoL in this study. Patients with comorbidities were nearly four times more likely to report low QoL (AOR = 3.79, p < 0.001). This finding is consistent with the literature indicating that comorbidity burden substantially reduces both physical and mental QoL among dialysis patients [11,17,24]. Feroze et al. highlighted the direct impact of multiple comorbidities on functional limitations and mental distress [11]. Integrated disease management strategies are thus crucial to improving overall health outcomes in ESRD.

Substance use

Substance use showed a significant association with poor QoL in the unadjusted analysis (OR = 2.08, p = 0.011), but the effect diminished after adjustment (AOR = 1.82, p = 0.052). This suggests that its impact may be confounded by variables such as socioeconomic status and comorbidity status. Kimmel et al. reported similar findings, where substance use, particularly alcohol and illicit drugs, was linked to lower psychosocial functioning among ESRD patients [18]. However, its independent effect often weakens after adjusting for other influencing factors [29]. Despite this, addressing substance use remains important in comprehensive CKD care models.

Dialysis frequency and duration of chronic kidney disease

Participants undergoing fewer than 100 dialysis sessions annually had significantly better QoL (AOR = 0.34, p < 0.001), indicating that more frequent dialysis sessions may improve outcomes. This aligns with studies by Mittal et al. and Anees et al., who demonstrated that greater dialysis exposure is associated with enhanced physical and mental functioning [13,16]. These findings highlight the importance of ensuring adequate dialysis frequency to alleviate symptom burden and support long-term well-being.

Limitations

This study had several limitations. Its cross-sectional design precludes causal inference or evaluation of temporal changes in QoL. Being a single-center study conducted in an urban tertiary care hospital, findings may not be generalizable to rural or lower-resource settings. QoL data were self-reported, which may introduce recall and social desirability biases. Additionally, psychological conditions such as depression and anxiety, important contributors to QoL, were not assessed using validated tools. Nutritional status, lifestyle factors, and social support systems, all of which influence QoL, were also not evaluated. Finally, the study excluded patients receiving peritoneal dialysis or conservative care, limiting comparative insights across CKD management modalities.

## Conclusions

This study highlights the significantly compromised QoL among CKD patients undergoing hemodialysis, with nearly half reporting low QoL scores. Key independent predictors of poor QoL included low socioeconomic status, presence of comorbidities, longer disease duration, and reduced dialysis frequency. Notably, patients receiving more than 100 dialysis sessions per year demonstrated better QoL, emphasizing the role of adequate treatment access and adherence. While demographic variables such as age and gender did not retain statistical significance in adjusted analyses, their influence on specific psychosocial domains may still be relevant and warrants further investigation. These findings underscore the need for multidisciplinary care strategies that address socioeconomic barriers, enhance comorbidity management, and ensure regular, accessible dialysis to improve health-related QoL among CKD patients.
